# Caregiving burdens of task time and task difficulty among paid and unpaid caregivers of persons living with dementia

**DOI:** 10.3389/fpubh.2025.1615187

**Published:** 2025-07-09

**Authors:** Matthew Lee Smith, Jodi L. Southerland, Malinee Neelamegam, Gang Han, Shinduk Lee, Chung Lin Kew, Juanita-Dawne R. Bacsu, Elyse Couch, Steffi M. Kim, Monique J. Brown, Ayse Malatyali, Lucas Wilson, Zahra Rahemi, Jeremy Holloway, Marcia G. Ory

**Affiliations:** ^1^School of Public Health, Texas A&M University, College Station, TX, United States; ^2^Center for Community Health and Aging, Texas A&M University, College Station, TX, United States; ^3^College of Public Health, East Tennessee State University, Johnson City, TN, United States; ^4^College of Public Health, The University of North Texas, Forth Worth, TX, United States; ^5^College of Nursing, The University of Utah, Salt Lake City, UT, United States; ^6^School of Nursing, Thompson Rivers University, Kamloops, BC, Canada; ^7^School of Public Health, Brown University, Providence, RI, United States; ^8^Department of Psychology, University of Anchorage, Anchorage, AK, United States; ^9^Arnold School of Public Health, University of South Carolina, Columbia, SC, United States; ^10^College of Nursing, University of Central Florida, Orlando, FL, United States; ^11^School of Nursing, Clemson University, Clemson, SC, United States; ^12^School of Education, New Mexico Highlands University, Las Vegas, NM, United States

**Keywords:** unpaid caregiver, paid caregiver, strain, mental health, caregiver burden, dementia

## Abstract

**Background:**

Demands of caregivers of persons living with dementia (PLWD) are often influenced by the context of their caregiving situation. This study examines common and unique factors associated with caregiving burden in terms of task time and task difficulty among paid and unpaid caregivers of PLWD.

**Methods:**

Cross-sectional baseline survey data were analyzed from 107 paid and unpaid caregivers of PLWD participating in a larger NIH-funded study assessing the feasibility of using a novel in-situ sensor system. Oberst Caregiving Burden Scale constructs of task time and task difficulty served as dependent variables. Two least squares regression models were fitted, controlling for contextual items related to the caregiver, care recipient, and caregiving logistics.

**Results:**

Caregivers whose care recipients were female (*B* = −0.29, *p* = 0.006), had more chronic conditions (*B* = 0.31, *p* = 0.011), and had lower Mini-Mental State Exam scores (*B* = −0.20, *p* = 0.015) reported higher task time burdens. Caregivers whose care recipients had other paid caregivers (*B* = 0.30, *p* = 0.031) and spent more months/years caring for their care recipients (*B* = 0.28, *p* = 0.004) reported higher task time burdens. Caregivers’ task time burden was positively associated with their emotional stress level (*B* = 0.30, *p* = 0.020). Caregivers’ task difficulty burden was positively associated with their emotional stress (*B* = 0.30, *p* = 0.029) and depressive symptomatology (*B* = 0.32, *p* = 0.002).

**Discussion:**

Results reinforce the relationship between caregiver burden and mental health impacts. While the care recipient’s disease profile and needs were drivers of task time burden, which may also require coordination with other paid caregivers, task difficulty was emotionally driven. Findings highlight the importance of caregiver support services and programming for mental health.

## 1 Introduction

Approximately 21% of Americans (53 million) provide unpaid care for a family member, friend, or neighbor (1). Of those, about half care for persons living with dementia (PLWD) (2). Beyond unpaid care, between 25 and 50% of community-dwelling PLWD also receive paid care (3). Given dementia caregiving is considered a chronic stressor (4) examining ways to address the burden of caring for PLWD is an important area of research (5).

Paid and unpaid caregivers are exposed to a challenging caregiving context that impacts their physical and psychological wellbeing (6, 7). However, the complex nature of dementia caregiving carries an added burden compared to non-dementia care environments. For example, caregiving tasks are more intense and require more time to perform for PLWD compared to other care recipients. PLWD also require more assistance with activities of daily living (ADL) and instrumental activities of daily living (IADL) and require assistance for a longer duration (8–11). Because of this high care burden context, caregivers of PLWD report significantly poorer physical and mental health outcomes than other caregivers (9–12).

Research on the care burden and demands of caregivers of PLWD has identified several key contributing factors. Functional decline and ADL-IADL performance progressively worsen as dementia progresses, thereby increasing levels of dependency on others (13–16). In a longitudinal study, the average hours of care per month for community-dwelling PLWD increased by 131 h over an eight-year period (17). The occurrence and severity of behavioral and neuropsychiatric symptoms (BNPS) also worsen as dementia progresses, which increases the complexity of care (14, 16, 18–21). The burden of caring for PLWD becomes even more complicated when the individual has other comorbid conditions (22). Several studies report that care tasks become more difficult and time consuming as the number and severity of chronic conditions increase (16, 22).

Overall, caregiver burden may be more pronounced among caregivers of female PLWD. When examining all-cause dementia, females exhibit faster decline in cognitive and executive functioning (23) and more neuropsychiatric symptoms such as delusions, hallucinations, and depression than men (24–28). Paradoxically, females also experience higher chronic disease and frailty burden, despite having lower mortality rates (29–31). Thus, female PLWD may require greater assistance for longer periods than males of the same age.

The demands of caregiving typically increase as duration of care extends. In contrast to adaptation theory, wear-and-tear theory posits that providing care for an extended period can have a negative impact on the caregiver (32). Prolonged caregiving can take a considerable toll on an individual and the cumulative effects of chronic stress and caregiver burden may erode an individual’s ability to provide care effectively. In a three-year longitudinal study, prolonged caregiving was associated with increased burden in caregivers without support (33). Moreover, because individuals with advanced dementia require more assistance, caregiver task difficulty and time may increase over time (20). Individuals may be more likely to seek paid caregiving support to ease the burden of care (34).

Most research confirms that high intensity care demands, such as ADL and IADL assistance, are associated with worse emotional and physical health outcomes for caregivers (7, 35, 36). For example, completing physically demanding care tasks, such as bathing, dressing, and transferring (7, 37, 38), is associated with increased risk of emotional, physical, and financial burden (39). Similar findings have been reported elsewhere (40, 41). However, a separate systematic review concluded that objective measures of care demands, such as number of tasks and assistance with ADLs or IADLs, do not always contribute to poorer quality of life (42), which underscores the variability in perceptions of the degree of difficulty of the care context among caregivers.

### 1.1 Measurement of caregiving burden

Caregiver burden is a multi-dimensional concept that comprises of multifactorial stressors including the emotional, physical, social, and financial strain of providing care to individuals with chronic illnesses (43). Most studies and measures look at general caregiver burden alone or in a single dimension, such as subjective or objective indicators of burden (44). Additionally, most U.S.-based studies use the Zarit Burden Interview, a measure of subjective experiences of burden to examine caregiver burden (45). More robust and targeted measures should be used to identify the unique domains, particularly related to objective indictors and sources of care-related burden (46). For example, subjective measures of care burden are more consistently associated with quality of life; whereas objective measures such as caregiving demands and time spent caring are not consistently related to health-related outcomes (42). While most studies examine time spent per day or duration of caregiving, less is known about the task time- and difficulty-related aspects of caregiving burden. The Oberst Scale enables the examination of how time and task difficulty influence caregiver burden (47), which can aid the investigation of objective indicators of burden.

Because time- and task-related difficulty contribute to emotional stress in caregivers (48), it is important to examine these dimensions of burden. Researchers contend that identifying the source of caregiver burden, specifically time- and difficulty-related care tasks that contribute to stress, is important for designing effective interventions (44, 47). By identifying the specific source of burden, practitioners can enhance intervention development and delivery (46). The purposes of this study were to identify the prevalence of caregiving burden among paid and unpaid caregivers of PLWD and examine the common and unique factors associated with caregiving burden in terms of task time and task difficulty.

## 2 Methods

### 2.1 Participants and procedures

Data were collected from paid and non-paid caregivers of PLWD as part of a larger Small Business Innovative Research Phase II grant funded by the National Institute of Aging (1R44AG065118-01). The objectives of the program were to remotely monitor device usage and real time location of PLWD in home and assisted living settings and analyze continuous sensor data in attempt to recognize activities of daily living (ADL) over an 18-month period. Caregiver and care recipient dyads were initially recruited into the study from assisted living facilities, home care entities, and home health companies. However, because of lock-down and visitation restrictions during COVID-19, recruitment from these settings was limited. Expanded recruitment strategies also included recruitment from healthcare facilities (e.g., physicians’ offices, pharmacies, and senior housing), Area Agencies on Aging, Meals on Wheels sites, and other community outreach (e.g., community presentations/tabling, flyers, newspaper articles, radio, social media).

Inclusion criteria for caregivers required that they: (a) be fluent in English to understand and sign consent documents; (b) have a high school diploma or equivalent; (c) be age 18 years or older; (d) provide care to care recipient with cognitive impairment at least 6 h per week; (e) willing to complete all requested questionnaires and checklists at baseline and at 9- and 18-month follow-ups; (f) plan to continue providing care to care recipient for the study duration; (g) not be pregnant; and (h) be willing to wear or carry a sensor tag or key fob while providing care to care recipient.

Once recruited and consented into the study, participants completed a series of questionnaires and assessments. This study utilizes the questionnaire administered to the paid and unpaid caregivers at baseline. Other than the care recipients’ Mini-Mental State Examination (MMSE) score collected at baseline, other data about the care recipient was reported by the caregiver. The caregiver baseline instrument included items about the caregiver’s sociodemographics, the care recipient’s sociodemographics (reported by the caregiver), caregiving situation and logistics (e.g., time and duration of care, living situation, relationship to care recipient), caregiver’s physical and mental health and self-care behaviors, caregiver’s perceptions about caregiving, and caregiver’s perceptions about the use of technology (i.e., in caregiving and non-caregiving contexts). Approval for this study was obtained from the Texas A&M University Institutional Review Board (#2019-0250F).

### 2.2 Measures

#### 2.2.1 Dependent variables

The dependent variables for this study were from the Oberst Caregiving Burden Scale (OCBS). The OCBS is a 15-item questionnaire used to measure 15 common caregiving tasks related to personal, direct, indirect, interpersonal, and support care (49, 50). For each task sub-scale, the measure allows participants to respond in two ways; rating the time related to the task and the difficulty associated with the task. Response choices for the time on task sub-scale use a 5-point Likert-type scale ranging from “none” (scored 1) to “a great amount” (scored 5). Time sub-scale scores range from 18 to 90, with higher scores indicating more time-related burden. Response choices for the task difficulty sub-scale use a 5-point Likert-type scale ranging from “not difficult” (scored 1) to “extremely difficult” (scored 5). Difficulty sub-scale scores range from 18 to 90, with higher scores indicating more difficulty-related burden. In the current sample, the internal reliability coefficients (i.e., Cronbach’s alpha) for the time and difficulty sub-scales were 0.918 and 0.932, respectively.

#### 2.2.2 Caregiver characteristics

Sociodemographic characteristics of the caregiver included in analyses were age and sex (i.e., male, female). Caregivers reported their relationship with their care recipient (i.e., spouse, paid caregiver, adult child, other relative/non-relative) and if they lived with their care recipient (i.e., no, yes). Caregivers also reported the duration in which they provided care to their care recipient [i.e., <3 months (scored 1), 3 to <6 months, 6 months to <1 year, 1 year to <2 years, 2 to <5 years, 5 to <10 years, 10+ years (scored 7)], which was treated continuously in analyses. Caregivers were also asked to report information about their physical and mental health. Variables included in this study were self-reported physical strain, emotional stress, and depressive symptomatology, each of which were treated continuously in analyses. For physical strain, participants were asked “How much of a physical strain would you say that caring for the care recipient is for you?” Response choices for this single item ranged from “not a strain at all” (scored 1) to “very much a strain” (scored 5). For emotional stress, participants were asked “How emotionally stressful would you say that caring for the care recipient is for you?” Response choices for this single item ranged from “not at all stressful” (scored 1) to “very stressful” (scored 5). For depressive symptomatology, participants were asked two items, “Over a typical 2-week period, how often have you been bothered by the following problems?: (a) Little interest or pleasure in doing things; and (b) feeling down, depressed, or hopeless.” Response choices for these items ranged from “not at all” (scored 0) to “nearly every day” (scored 3). Responses for these items were summed to create a composite score ranging from 0 to 6, with higher scores indicating more depressive symptomatology.

#### 2.2.3 Care recipient characteristics

Sociodemographic characteristics of the care recipient included in analyses were age and sex (i.e., male, female). Caregivers reported the number of chronic health conditions in which their care recipient had been diagnosed from a ‘check all that apply’ list of 15 conditions. Endorsed conditions were summed to create a count variable, which was treated continuously in analyses. Care recipients’ home environment included whether they resided in a single-family home, individual apartment or condominium, independent living community, or assisted living facility. Caregivers reported if their care recipient also had other paid caregivers (i.e., no, yes). At baseline, per the parent study protocol, a Mini-Mental State Examination (MMSE) was performed with all care recipients (51). The abbreviated MMSE is an 11-item cognitive function test administered in a paper-pencil format, which is among the most widely recognized tools for assessing cognitive states (52). Possible scores range from 0 to 30 (i.e., lower scores indicating more cognitive impairment) and was treated continuously in analyses.

### 2.3 Statistical analyses

All analyses were performed using SPSS (version 29). Descriptive statistics were calculated for all study variables, which were compared across unpaid and paid caregiver subgroups. OCBS composite scores, item-specific scores for time and difficulty sub-scales, were calculated and compared across unpaid and paid caregiver subgroups. Pearson’s Chi-square tests were used to identify proportional differences for categorical variables across caregiver groups. Independent sample t-tests were used to identify mean differences for continuous variables across caregiver groups. Two ordinary least squares (OLS) regression models were fitted to identify factors associated with OCBS time and difficulty sub-scales, respectively. The models were adjusted for characteristics of the caregiver, care recipient, and caregiving situation. Statistical significance was defined as *p* < 0.05.

## 3 Results

Baseline data were analyzed from 109 paid and unpaid caregivers of PLWD (Table 1). On average, the caregivers were age 58.87 (±15.76; range: 19–85) years. Most caregivers were female (78.4%), 56.0% lived with their care recipient, and 63.3% self-identified as unpaid caregivers. In terms of the relationship with their care recipients, 33.9% of caregivers reported being their spouse, 18.3% were their adult children, 11.0% were their other relatives, and 11.9% were non-relatives. About 62% of caregivers provided care to their care recipient for one or more years, with 26.6% providing care for five or more years. On average, caregivers reported providing 30.14 (±26.71) weekly hours of care to their care recipient. When comparing caregiver characteristics by caregiver type, on average, paid caregivers were younger than unpaid caregivers (i.e., 47.54 vs. 65.56, *t* = 6.77, *p* < 0.001) and cared for their care recipients for shorter durations (*t* = 2.62, *p* = 0.010). Relative to unpaid caregivers, smaller proportions of paid caregivers were male (i.e., 28.8% vs. 10.3%, *χ*^2^ = 4.92, *p* = 0.027) and lived with the care recipient (i.e., 81.2% vs. 12.5%, *χ*^2^ = 48.44, *p* < 0.001). Relative to unpaid caregivers, smaller proportions of paid caregivers were the spouse (i.e., 52.2% vs. 2.5%, *χ*^2^ = 27.87, *p* < 0.001) or adult child (i.e., 24.6% vs. 7.5%, *χ*^2^ = 4.96, *p* = 0.026) of their care recipients.

**Table 1:** Sample characteristics by caregiver type.

Variables	Total(*n* = 109)	Unpaid(*n* = 69)	Paid(*n* = 40)	*χ*^2^ or *t*	*p*
Caregiver characteristics					
Caregiving status					
Unpaid caregivers	63.3%	–	–		
Paid caregivers	36.7%	–	–		
Age (years) [range: 19–85]	58.87 (±15.76)	65.56 (±13.09)	47.54 (±13.32)	6.77	<0.001
Sex				4.92	0.027
Female	78.1%	71.2%	89.7%		
Male	21.9%	28.8%	10.3%		
Lives with their care recipient				48.44	<0.001
Yes	56.0%	81.2%	12.5%		
No	44.0%	18.8%	87.5%		
Relationship to recipient					
Spouse	33.9%	52.2%	2.5%	27.87	<0.001
Adult children	18.3%	24.6%	7.5%	4.96	0.026
Other relatives	11.0%	10.1%	12.5%	0.14	0.705
Non-relatives	11.9%	11.6%	12.5%	0.02	0.888
Hours care for recipient weekly [range: 0–100]	30.14 (±26.71)	33.00 (±29.23)	25.28 (±21.25)	1.58	0.117
Length of time cared for recipient [range: 1–7]	4.10 (±1.80)	4.43 (±1.78)	3.53 (±1.69)	2.62	0.010
Care recipient characteristics					
Age (years) [range: 64–100]	78.90 (±8.49)	78.06 (±8.07)	80.25 (±9.07)	−1.29	0.201
Sex				27.70	<0.001
Female	57.5%	37.9%	90.0%		
Male	42.5%	62.1%	10.0%		
Number of chronic conditions [range: 0–15]	2.08 (±2.96)	1.07 (±0.71)	3.87 (±4.31)	−4.02	<0.001
Living arrangement				24.20	<0.001
Single-family homes	65.7%	79.4%	42.5%		
Individual apartments or condominiums	17.6%	13.2%	25.0%		
Independent living community or assisted living facility	16.7%	7.4%	32.5%		
Has additional paid caregivers				11.30	<0.001
Yes	28.0%	17.6%	50.0%		
No	72.0%	82.4%	50.0%		
MMSE scores [range: 13–28]	22.60 (±2.67)	22.48 (±2.81)	22.79 (±2.44)	−0.56	0.579

When reporting on behalf of their care recipient, on average, caregivers reported their care recipients were age 78.90 (±8.49; range: 64–100) years and had 2.08 (±2.96) chronic health conditions. Most care recipients were female (57.5%) and resided in single-family homes (65.1%). An additional 17.4% resided in individual apartments or condominiums and 16.7% resided in independent living community or assisted living facility. On average, care recipients’ baseline MMSE score was 22.60 (±2.67; range: 13–28), with most falling in the mild cognitive impairment range. When comparing care recipient characteristics by caregiver type, a smaller proportion of paid caregivers cared for male care recipients (i.e., 62.1% vs. 10.0%, *χ*^2^ = 27.70, *p* < 0.001). On average, relative to unpaid caregivers, care recipients of paid caregivers had more chronic conditions (i.e., 1.07 vs. 3.87, *t* = −4.02, *p* < 0.001). Relative to unpaid caregivers, smaller proportions of paid caregivers had care recipients that resided in single-family homes (i.e., 79.4% vs. 42.5%, *χ*^2^ = 24.20, *p* < 0.001) and had additional paid caregivers for their care recipients (i.e., 82.4% vs. 50.0%, *χ*^2^ = 11.30, *p* < 0.001).

Table 2 reports the average scores for the OCBS sub-scale composite scores as well as average score for each sub-scale item, which are compared by caregiver type. On average, caregivers’ OCBS time sub-scale scores were 39.82 (±13.27) and their OCBS difficulty sub-scale scores were 25.62 (±11.25). OCBS time and difficulty sub-scale scores were significantly positively correlated (*r* = 0.60, *p* < 0.001). For the OCBS time sub-scale, the five tasks with the highest time-related burden were household tasks, emotional support, monitoring symptoms, transportation, and errands. For the OCBS difficulty sub-scale, the five tasks with the highest difficulty-related burden were behavior problems, emotional support, communicating with care recipient, household tasks, and communicating with healthcare professionals.

**Table 2:** OCBS sub-scale composite and item-specific scores by caregiver type.

Variables	OCBS time sub-scale					OCBS difficulty sub-scale				
	Total (*n* = 109)	Unpaid (*n* = 69)	Paid (*n* = 40)	*t*	*p*	Total (*n* = 109)	Unpaid (*n* = 69)	Paid (*n* = 40)	*t*	*p*
OCBS sub-scales (composite scores)	39.82 (±13.27)	39.12 (±13.62)	41.03 (±12.73)	−0.72	0.472	25.62 (±11.25)	28.04 (±11.42)	21.45 (±9.73)	3.20	0.002
OCBS sub-scale items										
Household tasks	3.48 (±1.34)	3.28 (±1.38)	3.83 (±1.20)	−2.10	0.038	1.83 (±1.04)	2.01 (±1.08)	1.53 (±0.91)	2.42	0.017
Emotional support	3.36 (±1.22)	3.12 (±1.17)	3.78 (±1.21)	−2.80	0.006	1.94 (±1.19)	2.20 (±1.24)	1.50 (±0.96)	3.30	0.001
Monitoring symptoms	3.27 (±1.23)	2.96 (±1.22)	3.80 (±1.07)	−3.64	<0.001	1.75 (±1.03)	1.87 (±1.04)	1.55 (±0.99)	1.57	0.119
Transportation	2.96 (±1.52)	3.29 (±1.43)	2.40 (±1.52)	3.07	0.003	1.75 (±1.16)	1.99 (±1.28)	1.35 (±0.77)	3.24	0.002
Errands	2.94 (±1.40)	3.07 (±1.39)	2.70 (±1.42)	1.34	0.183	1.68 (±0.96)	1.87 (±0.98)	1.35 (±0.83)	2.81	0.006
Behavior problems	2.74 (±1.40)	2.71 (±1.41)	2.80 (±1.40)	−0.32	0.748	2.06 (±1.26)	2.25 (±1.23)	1.75 (±1.26)	2.02	0.046
Planning activities	2.72 (±1.32)	2.63 (±1.23)	2.88 (±1.47)	−0.88	0.383	1.65 (±0.99)	1.84 (±1.02)	1.33 (±0.86)	2.81	0.006
Communicating with healthcare professionals	2.69 (±1.41)	2.78 (±1.31)	2.53 (±1.55)	0.91	0.366	1.76 (±1.08)	1.86 (±1.09)	1.59 (±1.07)	1.23	0.223
Communicating with care recipient	2.65 (±1.35)	2.54 (±1.32)	2.85 (±1.39)	−1.16	0.247	1.86 (±1.15)	2.09 (±1.16)	1.48 (±1.04)	2.76	0.007
Finances	2.44 (±1.55)	2.70 (±1.53)	2.00 (±1.50)	2.29	0.024	1.75 (±1.08)	1.94 (±1.11)	1.43 (±0.96)	2.46	0.015
Finding resources	2.42 (±1.36)	2.60 (±1.37)	2.10 (±1.30)	1.88	0.063	1.57 (±0.95)	1.77 (±1.00)	1.23 (±0.73)	3.25	0.002
Treatments (medications)	2.26 (±1.13)	2.01 (±0.96)	2.68 (±1.29)	−2.82	0.006	1.46 (±0.82)	1.54 (±0.82)	1.33 (±0.83)	1.30	0.198
Personal care	2.19 (±1.17)	2.00 (±1.13)	2.53 (±1.18)	−2.31	0.023	1.54 (±0.85)	1.59 (±0.86)	1.45 (±0.82)	0.86	0.393
Mobility	2.06 (±1.10)	1.87 (±1.01)	2.38 (±1.17)	−2.37	0.020	1.47 (±0.80)	1.54 (±0.85)	1.35 (±0.70)	1.24	0.220
Planning care while away	1.81 (±1.23)	1.78 (±1.09)	1.85 (±1.46)	−0.29	0.776	1.56 (±1.14)	1.72 (±1.21)	1.30 (±0.97)	1.99	0.050

When comparing the OCBS time sub-scale by caregiving type, no significant difference in the composite score was observed between unpaid and paid caregivers. On average, relative to paid caregivers, unpaid caregivers reported higher time burdens with transportation (*t* = 3.07, *p* = 0.003) and finances (*t* = 2.29, *p* = 0.024). Conversely, on average compared to unpaid caregivers, paid caregivers reported time burdens with household tasks (*t* = −2.10, *p* = 0.038), emotional support (*t* = −2.80, *p* = 0.006), monitoring symptoms (*t* = −3.64, *p* < 0.001), treatments (medications) (*t* = −2.82, *p* = 0.006), personal care (*t* = −2.31, *p* = 0.023), and mobility (*t* = −2.37, *p* = 0.020).

When comparing the OCBS difficulty sub-scale by caregiving type, unpaid caregivers reported higher average composite scores than paid caregivers (*t* = 3.20, *p* = 0.002). On average, relative to paid caregivers, unpaid caregivers reported higher difficulty burdens with household tasks (*t* = 2.42, *p* = 0.017), emotional support (*t* = 3.30, *p* = 0.001), transportation (*t* = 3.24, *p* = 0.002), errands (*t* = 2.81, *p* = 0.006), behavior problems (*t* = 2.02, *p* = 0.046), planning activities (*t* = 2.81, *p* = 0.006), communicating with the care recipient (*t* = 2.76, *p* = 0.007), finances (*t* = 2.46, *p* = 0.015), and finding resources (*t* = 3.25, *p* = 0.002).

Table 3 reports findings from the OLS regression model fitting caregivers’ task time burden. On average, caring for men, compared to women, was associated with lower task time burden (*β* = −0.29, *p* = 0.006). Caring for care recipients with higher baseline MMSE scores (*β* = −0.20, *p* = 0.015) and more chronic health conditions (*β* = 0.31, *p* = 0.011) was associated with higher task time burden. On average, caregivers who cared for their care recipient for longer durations (*β* = 0.28, *p* = 0.004) and whose care recipients also had other paid caregivers (*β* = 0.30, *p* = 0.031) reported higher task time burden. On average, higher emotional stress levels experienced by caregivers was also positively associated with higher task time burden (*β* = 0.30, *p* = 0.020).

**Table 3:** Factors associated with task time and task difficulty burdens (composite sub-scale scores).

Variables	OBCS task time burdens					OBCS task difficulty burdens				
				95% CI					95% CI	
	Beta	*t*	*p*	Lower	Upper	Beta	*t*	*p*	Lower	Upper
Caregiver age	−0.14	−1.23	0.223	−0.31	0.07	0.09	0.73	0.468	−0.11	0.24
Caregiver female (vs. male)	0.04	0.49	0.623	−4.00	6.63	0.09	1.03	0.309	−2.36	7.36
Care recipient female (vs. male)	−0.29	−2.83	0.006	−13.23	−2.31	0.03	0.32	0.753	−4.20	5.79
Care recipient number of chronic conditions	0.31	2.60	0.011	0.33	2.50	0.02	0.15	0.884	−0.92	1.07
Care recipient MMSE score	−0.20	−2.49	0.015	−1.82	−0.20	−0.08	−0.95	0.347	−1.09	0.39
Care recipient lives in single family home (vs. no)	0.14	0.87	0.390	−5.08	12.88	−0.10	−0.59	0.554	−10.67	5.76
Care recipient lives in individual apartment (vs. no)	0.07	0.48	0.633	−7.86	12.84	−0.19	−1.19	0.237	−15.14	3.80
Caregiver lives with care recipient (vs. no)	0.11	0.85	0.397	−4.02	10.01	0.01	0.06	0.952	−6.22	6.61
Caregiver is spouse to care recipient (vs. no)	−0.07	−0.54	0.594	−9.73	5.60	−0.03	−0.23	0.818	−7.83	6.20
Caregiver is child to care recipient (vs. no)	−0.01	−0.06	0.950	−7.14	6.70	0.02	0.17	0.863	−5.78	6.88
Caregiver is paid caregiver for care recipient (vs. unpaid)	−0.14	−0.99	0.325	−12.88	4.33	−0.21	−1.46	0.149	−13.64	2.11
Care recipient has other paid caregivers (vs. no)	0.30	2.20	0.031	0.78	15.55	−0.07	−0.47	0.642	−8.34	5.17
Duration caregiver has cared for care recipient	0.28	3.01	0.004	0.67	3.32	0.06	0.63	0.533	−0.83	1.59
Caregiver physical strain level	0.08	0.67	0.503	−1.81	3.67	0.05	0.44	0.661	−1.95	3.06
Caregiver emotional stress level	0.30	2.37	0.020	0.48	5.61	0.30	2.23	0.029	0.28	4.97
Caregiver depressive symptomatology level	0.15	1.53	0.130	−0.36	2.75	0.32	3.22	0.002	0.88	3.73
	Adjusted R Square = 0.486					Adjusted R Square = 0.428				

Table 3 also reports findings from the OLS regression model fitting caregivers’ task difficulty burden. On average, caregivers with higher emotional stress levels experienced higher task difficulty burden (*β* = 0.30, *p* = 0.029). On average, caregivers with higher depressive symptomatology levels experienced higher task difficulty burden (*β* = 0.32, *p* = 0.002).

## 4 Discussion

This study identified several factors associated with caregiving task time and task difficulty burden among paid and unpaid caregivers of PLWD (47, 48). Care recipients’ characteristics can affect the degree of burden experienced by unpaid and paid caregivers. Results from the present study reveal that task time burden was influenced by several factors including the sex, physical condition, and cognitive status of the care recipient. Research suggests that caring for men is generally more difficult than caring for women (53); however, in the present study, caring for men was associated with lower task time burden. Women have greater risk of functional impairment and cognitive decline than men (23, 54), factors that may contribute to higher ADL- and IADL-dependency. This may explain the higher task time burden associated with caring for women.

Findings also suggest that caregivers of PLWD with more chronic conditions and lower cognitive function report higher task time burden. The care dependent/time interaction is well-established in the literature. Specifically, low ADL and IADL levels increase caregiving time (13, 20, 22, 41). This care encompasses assistance with basic daily activities and ongoing medical care (55, 56), which can include frequent medications, symptom management, monitoring, and more care planning. Coordinating care with multiple healthcare providers, which is common for individuals with multiple chronic conditions (57), requires time-consuming effort to be allocated to care coordination and communication, contributing to the higher task time burden.

Task time burden reported by caregivers of PLWD with lower cognitive function can be due to the additional support and supervision that is required as the disease progresses. Dementia is a progressive disease, which results in declining independence and deteriorating cognitive function in the care recipient over time. Caregivers of PLWD in more advanced stages of the disease are often involved in providing constant supervision for the safety of the care recipients and may spend more time assisting with communication and interpreting the needs of their care recipients (17, 21, 58, 59).

Caregivers working with other paid caregivers and those providing longer duration of care also reported higher task time burden related to caregiving. Duration of care is among the strongest predictors caregiver burden (20, 44). While having a team of caregivers may intuitively appear to support caregivers, it can also require additional coordination among the caregivers. These additional time-consuming tasks include coordinating care tasks and schedules among caregivers, communicating key information across the caregiving team, and ensuring care services are consistent across the team (60). These tasks are often time consuming and when added to caregiving duty, it can result in burden associated with task time. It may also result in conflicts in the care team, which further increases the time spent resolving differences and disagreements.

In the current study, caregivers with higher emotional stress reported higher task time and task difficulty burden; whereas depressive symptomology contributed to higher burden related to task difficulty. Caregiving can have an intense emotional toll on the caregiver (7, 36, 61, 62). Among caregivers with pre-existing mental health conditions, the emotional burden from caregiving can be further amplified (63). Feelings of stress, fatigue and being overwhelmed from caregiving tasks can result from caregivers juggling the management of their own mental health while coping with the demands of caregiving, and having reduced resilience to cope with caregiving demands (64, 65). Additionally, there is a robust association between care burden and psychological distress among PLWD caregivers compared to individuals caring for stroke survivors or persons with frailty but no cognitive decline (66). Individuals with symptoms of depression are also more likely to face social isolation and lack of social support (67, 68). Given the importance of social support in managing caregiver burden, the lack of this can be a driving factor that increases the burden felt by caregivers with pre-existing mental health conditions (69).

In the current study, paid and unpaid caregivers reported differences in caregiving situations and caregiving burdens. Unpaid caregivers more frequently cared for individuals with fewer chronic comorbidities, but they were more likely to provide care within single-family homes, be the care recipients’ spouse or adult child, and provide care without additional paid caregiver support. While task time composite scores were similar across paid and unpaid caregivers, unpaid caregivers felt more time burden associated with managing logistics (i.e., transportation, finances) and paid caregivers expressed the time burdens associated with direct patient care (e.g., monitoring symptoms, treatment, personal care, mobility). These findings support previous literature about the unique challenges of unpaid caregivers to balance the time needed for caregiving duties, family responsibilities, and employment demands (1, 70). As such, while these tasks may not require large amounts of time per se, the time taken from other competing demands may cause feelings of burden among unpaid caregivers who are not being compensated for their time spent caregiving (1, 70). Unpaid caregivers reported higher task difficulty composite scores compared to paid caregivers. Unpaid caregivers generally reported more task difficulty managing logistics (e.g., household tasks, transportation, errands, finances), coordination (e.g., planning activities, finding resources), and interactions with their care recipient (e.g., emotional support, communicating with care recipient). Relative to paid caregivers, unpaid caregivers may perceive these tasks to be more difficult because they have not been formally trained to execute these tasks (71) or have limited finances to acquire caregiving support or resources (71). Further, unpaid caregivers may perceive tasks as more difficult because of the emotional distress related to the failing health or suffering of a loved one (72).

Study findings align with policy priorities of national organizations (73, 74), who recommend policy changes and strategies to support paid and unpaid caregivers in the United States. Reported difficulty associated with household tasks, errands, and transportation reinforce the need to build the national infrastructure of available, accessible, and affordable home- and community-based services (HCBS). These services can provide support to caregivers and provide them with respite opportunities to meet their own household logistics and emotional demands. Strategies to improve access and utilization of HCBS can include tax deductions for out-of-pocket care expenses, long-term care social insurance options, or paid family leave for employed caregivers (73). Expressed difficulty communicating with healthcare professionals highlights the need for productive and informed interactions between caregivers and the care recipients’ healthcare providers. Strategies to improve healthcare providers’ understanding about the unique caregiving circumstances of their care recipients include routinizing formal assessment of unpaid caregiving needs in clinical settings and incentivizing healthcare systems to incorporate unpaid caregivers into healthcare decision making processes (73). Considering the growing need for paid caregivers and the expanding caregiving workforce gap, dedicated efforts are needed to improve paid caregiver wages for HCBS for paid caregivers, expand training and vocational opportunities to grow the workforce, and establish standardized training and accreditation nationwide (73, 74).

### 4.1 Limitations

The present study is not without limitations. The study’s data were self-reported and participant responses may reflect social desirability and recall biases. The study sample was of modest size and consisted of both paid and unpaid caregivers. Therefore, despite identifying differences in caregiving situations and OCBS-related scores by caregiving paid/unpaid status, it is difficult to make strong generalizations regarding paid versus unpaid caregivers. Consequently, further research with larger and more diverse samples is necessary to understand caregiver task time and difficulty burden more thoroughly between paid and unpaid caregivers of PLWD. The sample consisted primarily of women making it difficult to conduct a comprehensive sex-based analysis of caregiver burden. This study did not control for potential confounding factors such as the caregivers’ history of depression, self-reported chronic conditions, or other aspects of wellness. Details pertaining to the caregiving tasks performed by the caregiver (based on the specific needs of the care recipient such as dressing, bathing, or incontinence tasks) were not uniformly collected in a robust manner, which limited the interpretation of the OCBS scores.

A strength of the current study was its novel use of the two-dimension OCBS, which is unique given its scoring can transcend unidimensional scales of caregiver burden or strain and contextualize findings in terms of task time and task difficulty. However, other more mainstream burden scales [e.g., the Zarit Caregiver Burden Scale (45)] should be used in concordance with the OCBS for consistency within the broader published literature and comparison to other studies. The OCBS sub-scales have no known clinical thresholds or scoring cut-points to indicate severity levels of caregiver burden. Future studies should attempt to determine such thresholds to make the OCBS clinically actionable in terms of risk identification and referral to programs and services. Additionally, future studies should attempt to utilize more objective measures of caregiving tasks and care recipient needs (e.g., use of wearables or sensors) to complement subjective self-reported measures.

## 5 Conclusion

This study examined factors associated with caregiving burden in terms of task time and task difficulty among paid and unpaid caregivers of PLWD. Our study’s results highlight the relationship between objective measures of caregiver burden and the impact on mental health. While the care recipient’s disease profile and needs were drivers of task time burden, which may also require coordination with other paid caregivers, task difficulty was emotionally driven. Caregiving burdens differed in terms of task time and task difficulty by the caregivers’ paid/unpaid status. Findings from our study have implications for clinicians, practitioners, and community organizations working to support caregivers of people living with dementia. More specifically, our study’s findings highlight the critical need for caregiver support services and mental health programming.

## Data Availability

The raw data supporting the conclusions of this article will be made available by the authors without undue reservation.
